# A profiling analysis of contributions of cigarette smoking, dietary calcium intakes, and physical activity to fragility fracture in the elderly

**DOI:** 10.1038/s41598-018-28660-y

**Published:** 2018-07-10

**Authors:** Thuy T. Pham, Diep N. Nguyen, Eryk Dutkiewicz, Jacqueline R. Center, John A. Eisman, Tuan V. Nguyen

**Affiliations:** 1 Engineering and Information Technology, University of Technology, Sydney, 2006 Australia; 2 Bone Biology Division, Garvan Institute of Medical Research, Sydney, 2006 Australia; 3 St Vincent Clinical School, UNSW, Sydney, Australia; 4 School of Public Health and Community Medicine, UNSW, Sydney, Australia; 5 Notre Dame University School of Medicine, Sydney, Australia

## Abstract

Fragility fracture and bone mineral density (BMD) are influenced by common and modifiable lifestyle factors. In this study, we sought to define the contribution of lifestyle factors to fracture risk by using a profiling approach. The study involved 1683 women and 1010 men (50+ years old, followed up for up to 20 years). The incidence of new fractures was ascertained by X-ray reports. A “lifestyle risk score” (LRS) was derived as the weighted sum of effects of dietary calcium intake, physical activity index, and cigarette smoking. Each individual had a unique LRS, with higher scores being associated with a healthier lifestyle. Baseline values of lifestyle factors were assessed. In either men or women, individuals with a fracture had a significantly lower age-adjusted LRS than those without a fracture. In men, each unit lower in LRS was associated with a 66% increase in the risk of total fracture (non-adjusted hazard ratio [HR] 1.66; 95% CI, 1.26 to 2.20) and still significant after adjusting for age, weight or BMD. However, in women, the association was uncertain (HR 1.30; 95% CI, 1.11 to 1.53). These data suggest that unhealthy lifestyle habits are associated with an increased risk of fracture in men, but not in women, and that the association is mediated by BMD.

## Introduction

Osteoporosis is a common metabolic bone disorder characterized by reduced bone strength and increased fracture risk. The disease affects more than 30% women and 15% men aged 50 years and older^1^. Bone strength is primarily reflected by bone mineral density (BMD), which is measured by the dual-energy X-ray absorptiometry (DXA) technology. The variation in BMD can account for up to 60% of the variance in bone strength^2^. Therefore, BMD is one of the most important predictors of fracture; each standard deviation (SD) reduction in BMD is associated with 2-fold increase in the risk of fracture^3^. Thus, osteoporosis has been operationally defined in terms of BMD^2,3^.

The variation in BMD is determined by multiple factors, including genetic and environmental factors^4,5^. Among environmental factors, lifestyle factors exert considerable effects on BMD^6–12^. Smoking is a key environmental risk factor because it is significantly associated with decreased hip BMD in old age^6^, but the effect of cigarette smoking on fracture is reversible^8^, A physically active lifestyle and stable weight are associated with reduced bone loss, and thus contribute to the reduction of fracture risk^11^. In the elderly, physical activity was associated with a 20–40% reduced risk of hip fracture (relative to sedentary subjects)^12^. Several studies have found that adequate dietary calcium intakes were associated with better bone health (i.e., higher bone mass and reduced fracture risk)^13,14^. Nevertheless, a recent meta-analysis has suggested that the effect of calcium intakes was uncertain^15^. Alcohol consumption has also been suggested as a relevant factor for bone health; however, the impact of alcohol consumption on bone health is complex and may be dependent on other factors^16^.

Earlier studies generally examined the effect of lifestyle factors individually. However, these factors tend to co-vary within an individual; smokers tend to have reduced physical health and athletic performance^17^, and individuals who exercise are less likely to smoke, and engaging in exercise may help quit smoking^18^. Similarly, adequate calcium intakes may maximize the positive effect of physical activity on bone health during the growth period of individuals^19^. Therefore, a more comprehensive and holistic approach may offer a better method to evaluate the magnitude of effects of multiple lifestyle factors on BMD or fracture burden.

We hypothesize that a linearly combined effect of low dietary calcium intake, cigarette smoking, and low physical activity is associated with a greater risk of fracture and lower BMD. The present study was designed to test the hypothesis by pursing the following two specific aims: (i) to quantify the contribution of each lifestyle factor to the variation in fracture risk; and (ii) to define the association between combined effects of lifestyle factors on fracture risk.

## Results

### Baseline characteristics

The study ultimately included 1960 women and 1170 men whose baseline characteristics are shown in Table 1. The average (SD) age of participants was 69 (6.18) years. The individuals’ health status had been followed monitored for between 0.1 and 20 years (average of 12 years). During the follow-up period, 597 women (30.5%) and 206 men (17.6%) had reported to sustaine a fragility fracture. Among of 803 fracture cases, 165 occurred at the hip and 331 at vertebrae in either women or men.

**Table 1:** Characteristics of individuals (baseline).

	Predictor	Unit	No fracture	Fracture	P^a^
			(mean, SD^b^)	(mean, SD)	
Men	N	people	963	206	
	Age	years	68.38 (6.18)	71.73 (6.73)	***
	Weight	kg	81.41 (13.74)	77.17 (12.21)	***
	Height	cm	173.79 (6.82)	172.26 (6.32)	**
	Body mass index	*kg*/*m*^2^	26.9 (3.99)	25.94 (3.79)	**
	Smoking	Packyears	21.14 (29.32)	30.06 (36.44)	***
	Smokers	Yes/No (n; %)	593 (61.58%)	138 (66.99%)	
	Max alcohol intakes	drinks/week^c^	1.33 (2.34)	1.54 (3.70)	
	Dietary calcium intakes	g/day	699.12 (358.85)	621.01 (377.11)	**
	Physical activity index	hours/week	33.27 (5.43)	32.47 (5.60)	
	Lumbar spine BMD^d^	*g*/*cm*^2^	0.94 (0.14)	0.86 (0.16)	***
	Femoral neck BMD	*g*/*cm*^2^	1.28 (0.21)	1.17 (0.20)	***
Women	N	people	1362	597	
	Age	years	67.78 (6.95)	70.96 (7.27)	***
	Weight	kg	68.51 (13.39)	64.88 (13.03)	***
	Height	cm	160.47 (6.09)	159.08 (6.69)	***
	Body mass index	*kg*/*m*^2^	26.61 (5.04)	25.59 (4.9)	***
	Smoking	Packyears	6.54 (14.81)	7.98 (16.31)	*
	Smokers	Yes/No (n; %)	406.00 (29.81%)	194.00 (32.50%)	***
	Max alcohol intake	drinks/week^c^	0.41 (0.78)	0.35 (0.8)	
	Dietary calcium intakes	g/day	718.85 (394.59)	689.58 (376.85)	
	Physical activity index	hours/week	30.75 (2.89)	30.41 (3)	*
	Lumbar spine BMD	*g*/*cm*^2^	0.83 (0.14)	0.75 (0.13)	***
	Femoral neck BMD	*g*/*cm*^2^	1.09 (0.20)	0.98 (0.19)	***

^a^Significane codes: ‘***’ is <0.001; ‘**’ 0.01; ‘*’ 0.05; ‘.’ 0.1; ‘’ >0:1.

^b^SD: standard deviation.

^c^Alcohol consumption intakes were assessed by standard drinks per week. 1 standard drink = a glass of wine or a middie of beer or a nip of spirits.

^d^BMD: Bone mineral density.

As expected, men and women with a fracture were older than those without a fracture. Individuals with fracture also had lower body weight, lower body mass index, lower BMD at the lumbar spine (LSBMD) or femoral neck (FNBMD). In men, fracture cases had significantly lower dietary calcium intakes and higher cigarette intakes (pack-years) than those without a fracture. In women, physical activity index was significantly lower in the fracture group compared with the non-fracture group.

### Lifestyle factors and BMD

**Figure 1:**
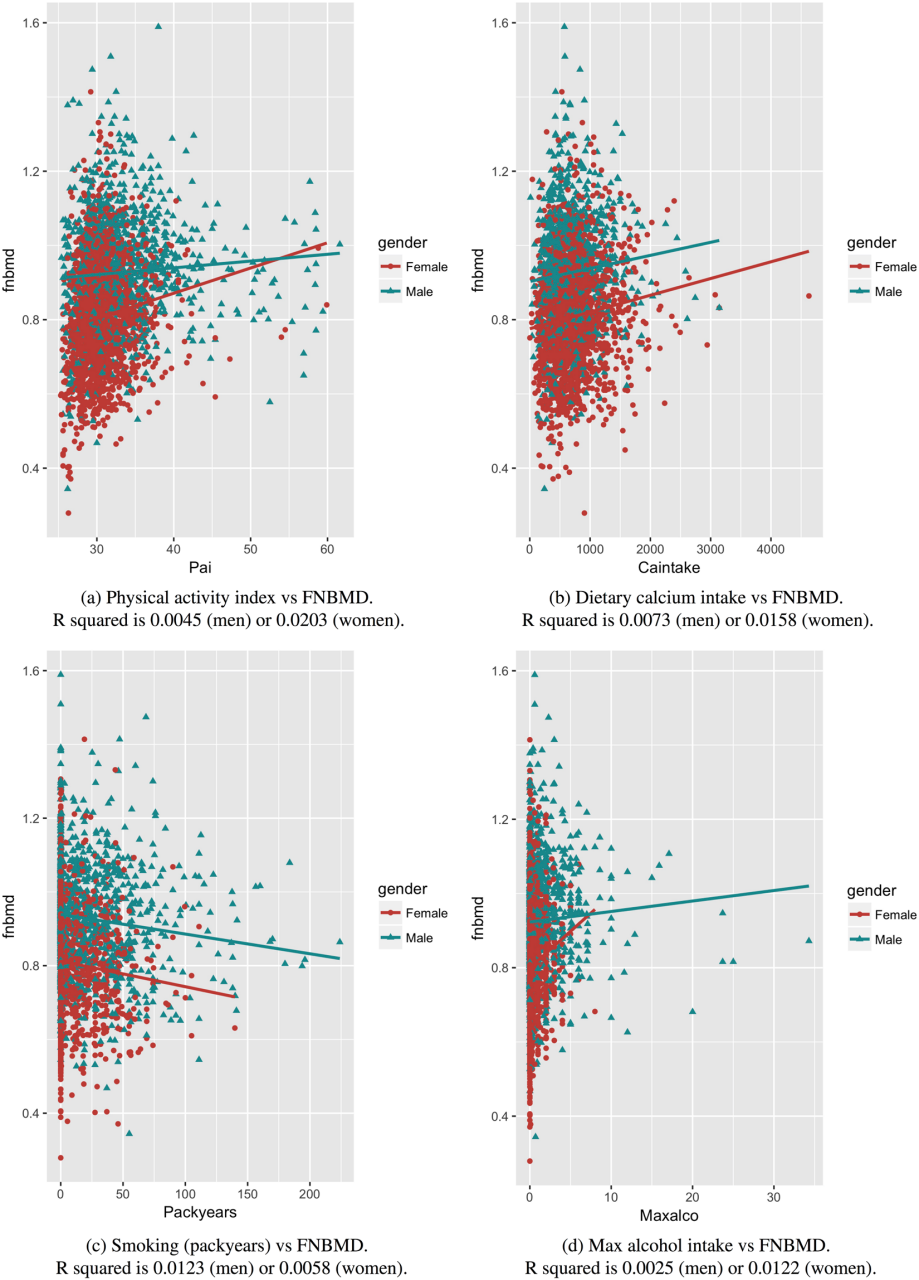
Lifestyle factors against femoral neck bone mineral density (FNBMD). R squared values are reported from corresponding linear regression estimations. The models were note adjusted for age and BMI. (**a**) Physical activity index vs FNBMD. R squared is 0.0045 (men) or 0.0203 (women). (**b**) Dietary calcium intake vs FNBMD. R squared is 0.0073 (men) or 0.0158 (women). (**c**) Smoking (packyears) vs FNBMD. R squared is 0.0123 (men) or 0.0058 (women). (**d**) Max alcohol intake vs FNBMD. R squared is 0.0025 (men) or 0.0122 (women).

Figure 1 shows the relationship between individual lifestyle factors and femoral neck BMD. In either men or women, greater dietary calcium intakes, and greater alcohol intakes were each associated with higher femoral neck BMD. Moreover, greater tobacco intakes were associated with lower femoral neck BMD. The magnitudes of association between each of the factors and femoral neck BMD or lumbar spine BMD are shown in terms of regression coefficients (Table 2).

**Table 2:** Lifestyle determinants of BMD using multiple linear regression analysis.

Predictor	Femoral neck (FN) BMD^a^		Lumbar spine (LS) BMD	
	Coefficient ± SE^b^	P-value^b^	Coefficient ± SE	P-value
**Men**				
Dietary calcium intakes	0.013 ± 0.005	0.003	0.029 ± 0.007	<0.001
Physical activity index	0.008 ± 0.003	0.022	−0.007 ± 0.005	0.166
Smoking Packyears	−0.013 ± 0.003	<0.001	−0.016 ± 0.005	0.001
Max alcohol intake	0.005 ± 0.003	0.088	0.001 ± 0.004	0.744
LRS^c^	0.085 ± 0.017	<0.001	0.097 ± 0.017	<0.001
**Women**				
Dietary calcium intakes	0.017 ± 0.003	<0.001	0.007 ± 0.004	0.119
Physical activity index	0.029 ± 0.005	<0.001	0.013 ± 0.007	0.044
Smoking Packyears	−0.016 ± 0.005	0.001	−0.005 ± 0.007	0.445
Max alcohol intake	0.035 ± 0.007	<0.001	0.045 ± 0.010	<0.001
LRS^c^	0.088 ± 0.010	<0.001	0.089 ± 0.035	0.012

^a^BMD: Bone mineral density. LRS: Lifestyle Risk Score.

^b^SE: standard error. P-value is for the coeffiecients in the linear regression analysis.

^c^I(LRS/0.1). Notes: Lifestyle predictors are normalized using zscores. Coefficients of linear regression are adjusted by age and body mass index.

**Figure 2:**
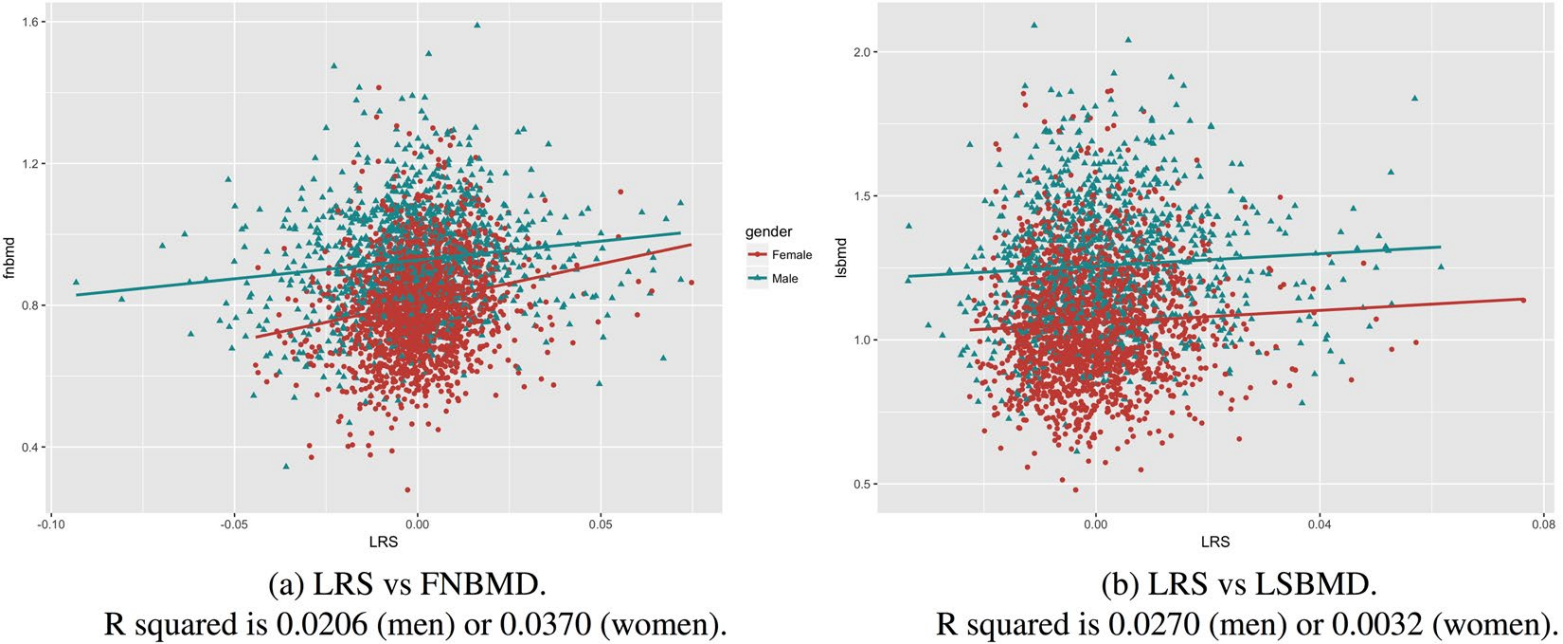
Lifestyle Risk Score (LRS) against femoral neck BMD (FNBMD) and lumbar spine BMD (LSBMD). R squared values are reported from corresponding linear regression estimations. The models were note adjusted for age and BMI. (**a**) LRS vs FNBMD. R squared is 0.0206 (men) or 0.0370 (women). (**b**) LRS vs LSBMD. R squared is 0.0270 (men) or 0.0032 (women).

When physical activity, dietary calcium intakes and smoking (packyears) were combined into a “lifestyle risk score” (LRS) by using the regression coefficients, greater LRS was significantly associated with higher *FNBMD* (Fig. 2a) and *LSBMD* (Fig. 2b) in men and women. In the linear regression analysis (Table 2) greater dietary calcium intakes, greater physical activity index, and higher alcohol intakes were each significantly associated with greater femoral neck BMD in men and women. We found that each unit increase in LRS was associated with 0.085 *g*/*cm*^2^ (*P* < 0.0001) greater femoral neck BMD in men or 0.088 *g*/*cm*^2^ (*P* < 0.0001) in women. The association between LRS and lumbar spine BMD remained statistically significant after adjusting for age and weight (Table 2). Moreover, greater tobacco intakes were associated with lower femoral neck BMD.

### Lifestyle factors and fracture

**Figure 3:**
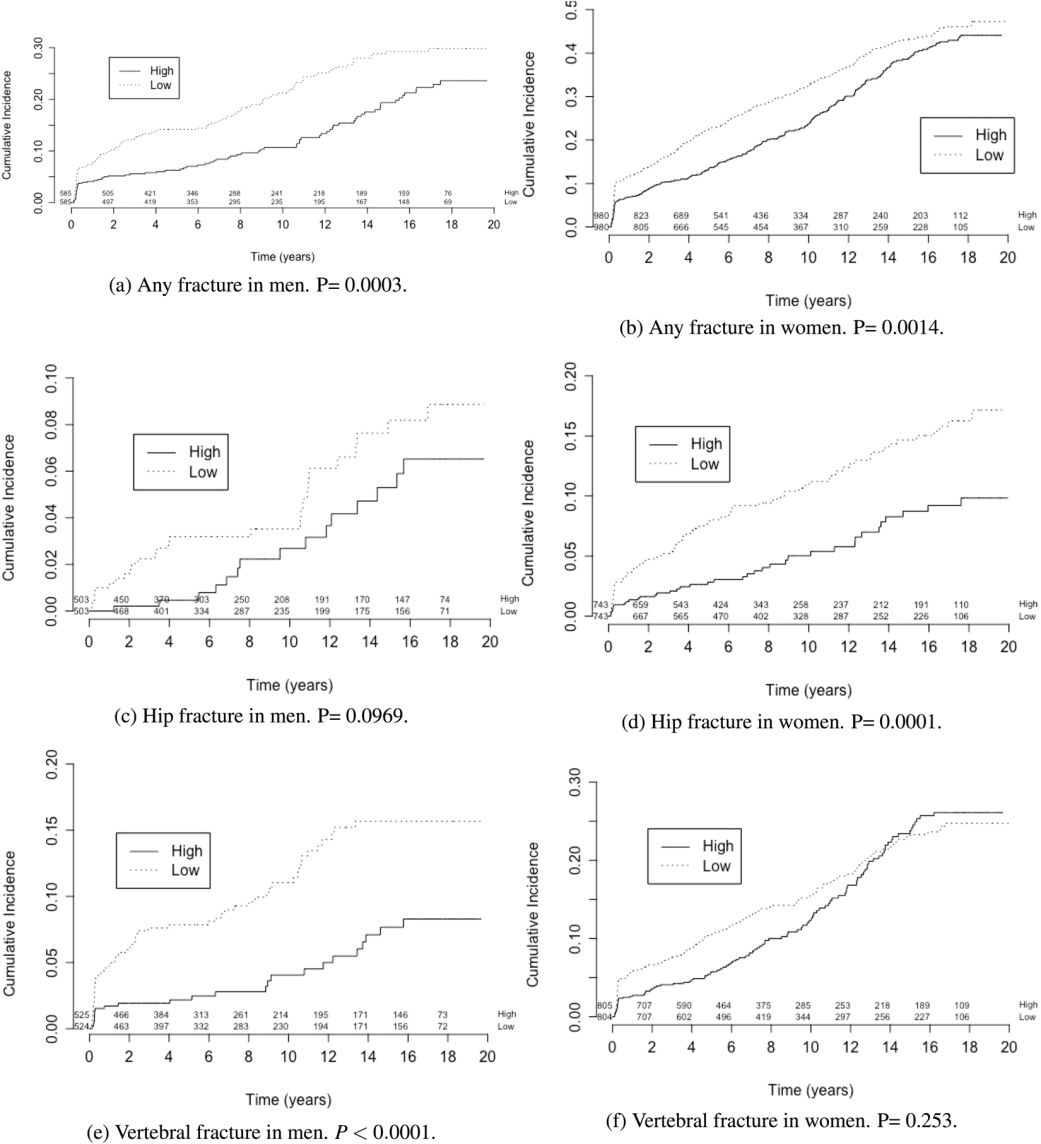
Cumulative Incidence over time (years) in men and women. P: P-value reported in each subplot is for the difference between survival curve by Chisquare test. (**a**) Any fracture in men. P = 0.0003. (**b**) Any fracture in women. P = 0.0014. (**c**) Hip fracture in men. P = 0.0969. (**d**) Hip fracture in women. P = 0.0001. **(e**) Vertebral fracture in men. *P* < 0.0001. (**f**) Vertebral fracture in women. P = 0.253.

For the ease of interpretation, we classified LRS into “low” (*LRS* < 0) or “high” (*LRS* ≥ 0) based on the median value. Individuals with low LRS had a greater risk of fracture than those with high LRS, and the difference was more pronounced in men (Fig. 3a) than in women (Fig. 3b). The association between LRS and fracture risk was also observed for hip fracture in men (Fig. 3c) and in women (Fig. 3d), for vertebral fracture in men (Fig. 3e) and in women (Fig. 3f).

Table 3 shows that low-LRS men had a 66% increase in the hazard of total fracture (HR: 1.66; 95% confidence interval (CI), 1.26 to 2.20). This association in men was more evident in for vertebral fracture (HR: 2.52; 95% CI, 1.57 to 4.05, *P* < 0.001), but not for hip fracture (HR: 1.70; 95% CI, 0.90 to 3.19, *P* = 0.1). In women, lower LRS was associated with a 30% increase in the hazard of total fracture (HR: 1.30; 95% CI, 1.11 to 1.53, *P* = 0.002), but the association was more pronounced for hip fracture (HR: 1.91; 95% CI, 1.31 to 2.79, *P* = 0.001). After adjusting for age and weight or BMI (Table 4), the HR of fracture at any site was 1.61 (95% CI, 1.22 to 2.13, *P* = 0.001) for men and 1.13 (95% CI, 0.96 to 1.33, *P* = 0.155) for women.

**Table 3:** Prediction of fracture risk using univariate Cox Proportional Hazards Analysis.

Fracture Site	Predictor^a^	Unit	Men		Women	
			HR^b^ (CI)	P-value^c^	HR (CI)	P-value
Any fracture	LRS	Low/High	1.66 (1.26–2.20)	<0.001	1.30 (1.11–1.53)	0.001
	Age	(+5 years)	1.52 (1.38–1.69)	<0.001	1.35 (1.28–1.43)	<0.001
	FNBMD^d^	(−0.12 *g*/*cm*^2^)	0.64 (0.56–0.72)	<0.001	0.60 (0.55–0.64)	<0.001
Hip fracture	LRS	Low/High	1.70 (0.90–3.19)	0.101	2.10 (1.43–3.07)	<0.001
	Age	(+5 years)	1.92 (1.53–2.40)	<0.001	1.93 (1.73–2.16)	<0.001
	FNBMD	(−0.12 *g*/*cm*^2^)	0.31 (0.23–0.42)	<0.001	0.30 (0.25–0.35)	<0.001
Vertebral fracture	LRS	Low/High	2.52 (1.57–4.05)	<0.001	1.16 (0.90–1.49)	0.253
	Age	(+5 years)	1.58 (1.35–1.86)	<0.001	1.48 (1.36–1.61)	<0.001
	FNBMD	(−0.12 *g*/*cm*^2^)	0.48 (0.40–0.58)	<0.001	0.50 (0.44–0.57)	<0.001

^a^LRS: Lifestyle Risk Score. LRS in Low/High. Age in (+5 years). Weight in (kg). BMI in (*kg* = *m*^*2*^). FNBMD: Femoral neck bone mineral density.

^b^HR: Hazard ratio. 95% CI: Confidence intervals of the hazard ratio.

^c^P-value: Global statistical significance of the model.

^d^FNBMD: Femoral neck bone mineral density.

**Table 4:** Hazard ratio of fracture associated with lifestyle profiling after adjusting for age, body mass index (BMI), femoral neck BMD, and prior fracture.

Fracture Site	LRS^a^ adjusted by	Men		Women	
		HR^b^ (CI)	P-value^c^	HR (CI)	P-value
Any fracture	Age	1.59 (1.20–2.11)	0.001	1.12 (0.95–1.32)	0.187
	Age + BMI	1.59 (1.20–2.11)	0.001	1.13 (0.96–1.33)	0.148
	Age + FNBMD	1.49 (1.13–1.98)	0.005	1.08 (0.91–1.27)	0.387
	Age + Weight	1.60 (1.21–2.12)	0.001	1.14 (0.96–1.34)	0.131
	Age + Priorfx	1.60 (1.21–2.12)	0.001	1.11 (0.94–1.31)	0.208
Hip fracture	Age	1.59 (0.84–2.99)	0.151	1.33 (0.89–1.97)	0.161
	Age + BMI	1.62 (0.86–3.04)	0.136	1.44 (0.97–2.15)	0.070
	Age + FNBMD	1.20 (0.63–2.29)	0.573	1.19 (0.80–1.77)	0.386
	Age + Weight	1.63 (0.87–3.07)	0.129	1.43 (0.96–2.13)	0.075
	Age + Priorfx	1.62 (0.86–3.05)	0.137	1.31 (0.88–1.96)	0.178
Vertebral fracture	Age	2.36 (1.47–3.78)	<0.001	0.94 (0.73–1.22)	0.652
	Age + BMI	2.36 (1.47–3.78)	<0.001	0.94 (0.73–1.22)	0.750
	Age + FNBMD	2.37 (1.47–3.80)	0.004	0.96 (0.74–1.24)	0.493
	Age + Weight	2.36 (1.47–3.78)	<0.001	0.94 (0.73–1.22)	0.821
	Age + Priorfx	2.35 (1.47–3.78)	<0.001	0.97 (0.75–1.26)	0.636

^a^LRS: Lifestyle Risk Score. LRS in Low/High. Age in (+5 years). Weight in (kg). BMI in (*kg* = *m*^*2*^). FNBMD: Femoral neck bone mineral density.

^b^HR: Hazard ratio. 95% CI: Confidence intervals of the hazard ratio.

^c^P-value: Global statistical significance of the model.

## Discussion

Osteoporosis can be considered a lifestyle disease because its susceptibility is linked to unhealthy lifestyle. Cigarette smoking, lack of physical activity, and low dietary calcium intakes are each associated with increased risks of osteoporosis and fracture. However, the association between individual lifestyle factors and fracture risk is not always consistent, due largely to sample size issue, and more importantly, to the covariation between lifestyle factors. In this study, we recognized the covariation by creating a profile for each individual, and we found that individuals with an “unhealthy” lifestyle profile had lower BMD and higher risk of fracture than those with a better healthy lifestyle. This finding deserves further elaboration.

It has long been known that lifestyle factors contribute to the development of osteoporosis and increased risk of fracture. Among lifestyle factors that are associated with osteoporosis, cigarette smoking is a prominent risk factor. Cigarette smoking was first linked to osteoporosis more than 40 years ago^20^. In a meta-analysis of 29 studies, postmenopausal bone loss was greater in current smokers than non-smokers, with 6% difference in BMD being observed by the age of 80 years old^21^. In women, the cumulative risk of hip fracture is 7% and 15% higher in current smokers than non-smokers aged 85 and 90 years, respectively^21^. In a recent meta-analysis, compared with non-smokers, the risk of osteoporotic fracture was 32% higher in smoking men and women^22^. In another study, the risk of hip fracture was 1.6-fold higher in men with history of smoking than those without, and cessation of smoking reduced the risk of hip fracture by 27%^23^. Our present result of the association between cigarette smoking and fracture or BMD further confirms the association reported in the literature.

The association between dietary calcium intakes and bone health is controversial. In this study, we also found that higher dietary calcium intakes were associated with greater BMD and reduced fracture risk, and this association was independent of age and body weight. Our finding is not necessarily consistent with previous studies. Indeed, the association between dietary calcium intakes and BMD or fracture is not always evident. In the Study of Osteoporotic Fractures, milk drinking and dietary calcium intake as estimated by the food-frequency questionnaires, were not significantly associated with fracture^24^. Actually, when compared with the never users, current users of calcium supplements were at greater risk of hip fractures^24^. A population-based study found that the association between dietary calcium intake and fracture risk was not linear^25^. A recent meta-analysis of seven randomised trials found no significant effect of calcium intake on hip fracture risk^26^. Taken together, although calcium is an important factor in bone health, the association between dietary calcium intake and bone density or fracture is less clear in observational studies.

Apart from smoking and dietary calcium intakes, we found that greater physical activity was associated with greater BMD and lower fracture risk. This finding is consistent with the literature^27,28^. A recent meta-analysis with 22 prospective cohort studies found that individuals with higher level of physical activity had a 30–40% lower risk of fracture compared to those who had little or no physical activity^27^. The beneficial effect of physical activity on bone health was also evident in a clinical trial in which the risk of overall fracture was 50% lower in the exercise group than the control^28^.

Whether alcohol consumption has a positive or negative influence on bone health is a contentious issue. In this study, we observed that, to estimate BMD, the maximum alcohol consumption tends to have a non-linear relationship with bone mineral density, as the linear coefficient (against either FNBMD or LSBMD) we found is not consistent for both men and women. Generally, lower alcohol intakes seemed to have positive influence on bone mineral density, but higher alcohol intakes tended to be associated with lower bone mineral density, but the relationship was not consistent for men and women. This observation seems be supported in by earlier works which suggested that the association between the maximum alcohol consumption and BMD or fractures is complex and even may be dependent on other factors^16^. Because of this complex relationship, we did not consider alcohol intakes in the lifestyle profiling in this study.

Cigarette smoking, dietary calcium intakes, and physical activity index are tend to be correlated. The co-occurrence of smoking and physical inactivity has been observed in at least 50 studies^29,30^, with smoking being associated with lower levels of physical activity^29^. Furthermore, smoking individuals with low physical activity index tend to be associated with lower dietary calcium intakes. Therefore, it is logical to consider that these factors simultaneously, even though individually some factors may not be associated with bone health. In this study, we have created a “lifestyle signature” or LRS for each individual based on the magnitude of association between these factors and BMD, and then related LRS with fracture risk. The adverse association between LRS and fracture risk indicates that the cumulative exposure to unhealthy lifestyle had detrimental effect on bone health in both men and women.

Our findings also have important implications for public health and clinical assessment. As lifestyle factors are modifiable, this association implies that improving lifestyle at the individual level can reduce fracture risk. At present, fracture risk assessment is based on clinical risk factors, with modest to good discrimination^31^. This study showed that the incorporation of lifestyle profiling into fracture risk assessment could improve the accuracy of fracture risk prediction, especially for vertebral fractures in men where discrimination based on clinical risk factors is modest.

The present findings must however be considered within the context of strengths and weaknesses of the study. The study was based on a large and well-characterized cohort, with long duration of follow-up that allows better detection of fine associations. We consider that our lifestyle profiling approach is a strength of the study, because with this approach we could simultaneously examined the impact of multiple risk factors on fracture. The ascertainment of fractures by X-ray, not self-reports, represents a strength of our study, because it avoided the problem of misclassification or misdiagnoses. Nevertheless, data concerning lifestyle factors in this study were self-reported, and misclassification and measurement error could be present in the analysis. The dietary calcium data were derived from a food frequency questionnaire with the time-frame of one week which may not reflect the long-term intake. We considered three lifestyle factors in this study, and we therefore could not exclude unmeasured confounding as partial accounting for the findings. Moreover, because the study was an observational investigation, no causal inference could be made on the relationship between lifestyle and fracture.

In conclusion, these data suggest that cumulative exposure to unhealthy lifestyle factors (e.g., smoking, lower physical activity index, and lower dietary calcium intakes) is associated with increased fracture risk, and the association is mainly driven by bone mineral density. Given that lifestyle risk factors are modifiable and that fracture prevalence is high in the general population, the present finding implies that the burden of fracture can be reduced at the individual level.

## Methods

### Study Design and Setting

This work is part of the Dubbo Osteoporosis Epidemiology Study (DOES), in which the protocol and study design have been described elsewhere^32,33^. Briefly, the Study involved 1581 men and 2095 women who were over 50 years old in Dubbo city and the surrounding districts (NSW, Australia) and were invited to participate in the Study in 1989. After missing data were excluded, data from 1683 women and 1010 men were analysed further. Dubbo city is located approximately 400 km north-west of Sydney. The city was chosen for this longitudinal study because of several reasons. First, it had population structure which resembled the overall Australian population. Second, its health services are largely self-contained allowing for the ascertainment of health outcomes, which made it become ideal for a prospective epidemiology study. More importantly, the city has 3 radiologists making it ideal for collecting almost all fractures within the area.

At the initial of the study in 1989, the population of Dubbo consisted of approximately 32,000 people, of whom approximately 99% were Caucasian and about 1% were Australian Aboriginal. All 1581 men and 2095 women aged 60 years or above as at mid-1989 residing in Dubbo and the two surrounding districts (Geurie and Wongarbon) which had the same postcode as Dubbo were invited to participate in the study. This study represented for 11% of the entire Dubbo population. Potential participants were chosen from the electoral roll (where enrolment is compulsory in Australia) and media campaigns. The rate of participation was 69% for women and 58% for men. Although all eligible men and women within the Dubbo area were invited to participate into the study, all study participants were of Caucasian origin. During follow-up, more participants were collected; however, majority of participants were recruited within the first two years of the commencement. The study’s procedure and protocol were approved by the St Vincent’s Campus Research Ethics Committee (NSW, Australia), and written informed consent was obtained from each participant. All research in this work were performed in accordance with relevant guidelines/regulations.

### Fracture Ascertainment

Non-trauma fracture was the main outcome of this study. The incidence of new fractures was ascertained through X-ray reports (provided by the two local radiology centres, as previously^34^). The fracture incidence had been continuously recorded since 1990. Fractures were included only if the report of fracture was definite and occurred with low trauma (e.g., fall from standing heights or less) which was confirmed on interview. Fractures were excluded from analysis if they were resulted from major trauma (e.g., motor vehicle accident), underlying diseases (e.g., cancer or bone-related diseases), or were fractures of the digits, skull, or cervical spine.

### Bone Mineral Density

Bone mineral density (BMD − *g*/*cm*^2^) measurement was performed by dual-energy X-ray absorptiometry (DXA) using a DPX densitometer (GELunar Corp., Madison, WI, USA) in the lumbar spine or femoral neck (radiation dose was <0.1 *pLGy*). Coefficient of variation for BMD in our normal subjects is 1–5% for lumbar spine and 1–3% for femoral neck^34^.

### Physical activity

The quantification of physical activity was based on the method used in the Framingham Massachusetts Heart Study^35^. Five levels of activities were considered: basal activity (sleeping or lying down), sedentary (sitting or standing), light (casual walking) moderate (gardening or carpentry), and heavy (lifting or heavy gardening). For each activity level, individuals were asked the average number of hours per week they spent performing each level of activity. The number of hours for each level was then translated to the average amount of oxygen consumed using the Metabolic Equivalent Task (MET) method. MET was derived as the product of the number of hours spent on each level of activity and an intensity weighting factor. The intensity factors for the levels were as follows: 1 = basal, 1.1 = sedentary, 1.5 = light, 2.4 = moderate, and 5 = heavy.

### Dietary calcium intakes

Dietary calcium intakes were assessed from a 4-day food frequency questionnaire^36^. The amount of dietary calcium intake for each individual was estimated as the sum of calcium content for each food item. The food items included daily intake of milk, bread, yogurt, cheese, ice cream, eggs, fish, cereal foods, fruits and vegetables, alcohols, and others (e.g., chocolate and orange juice). Since milk provides the highest amount of calcium in the diet, multiple questions relating to milk consumption were included to refine the estimate of dietary calcium intakes. The calcium content for each food item was derived from manufacturer’s information or from Australian or British tables of food consumption^36^.

### Lifestyle Factors

Alcohol intakes were assessed by the number of standard drinks (*g*/*day*). For example, a middie standard of beer, a standard glass of wine, or a nip of spirits equals to 10 *g*. Smoking was recorded as the number of cigarettes smoked per day, then the cumulative exposure was determined as the number of packyears in our analysis. Dietary calcium intakes were estimated as *mg*/*day*. Physical activity was quantified by the metabolic equivalent task (MET) which is the sum of products of the time spent on each activity level and a weighting factor derived from estimated oxygen consumption (i.e. sleep = 1.0, sedentary = 1.1, light activity = 1.5, moderate activity = 2.4, heavy activity = 5.0). The activities considered in the study were the number of hours of sleep per night, the number of hours spending on writing, reading, sitting, shower, grooming, cleaning, laundry, shopping, gardening, mowing lawn, manual labour digging, recreational activities (e.g., fishing, playing musical instrument, dance, or jogging), and occupational activities.

### Lifestyle Profiling

We constructed a single score called “lifestyle risk score” (LRS) that represented a lifestyle profiling for an individual. LRS is a linear summation of effects of smoking (packyears), dietary calcium intake, and physical activity index as follows.

1 $$LRS={\alpha }_{smoking}\times packyears+{\alpha }_{pai}\times pai+{\alpha }_{ca}\times Calcium$$

where *packyears* is smoking consumption, *pai* is physical activity index, and *Calcium* is the amount of dietary calcium intakes and their linear regression coefficients used to estimate BMD: *α*_*smoking*_, *α*_*pai*_, and *α*_*ca*_, respectively. LRS was normalized by z-score so that it has zero mean and unit variance.

### Data Analysis

We first used the multiple linear regression model to evaluate the association between lifestyle factors and BMD. The model was adjusted for age and body mass index because the two covariates are known to be associated with BMD. We then used the Cox’s proportional hazards model^37^ to evaluate the relationship between LRS and fracture risk. In the multivariable model, the hazard of fracture at time *t*, *H*(*t*), was modeled as a function of background risk, *H*_0_(*t*), and effects of LRS and covariates X: *H*(*t*) = *H*_0_(*t*) × *exp*(*βX*) where *β* is the regression coefficient of *X* to estimate fracture. Since age and femoral neck BMD are associated with fracture risk^38,39^, these two factors are considered covariates in the multi-variable analysis with Cox’s model. The magnitude of association between LRS and fracture risk was assessed by the hazard ratio (HR) and its 95% confidence interval (CI).

### Data Availability

The datasets generated during and/or analysed during the current study are available from the corresponding author on reasonable request.
